# Repeatability of artificial gravity tolerance times

**DOI:** 10.3389/fphys.2025.1464028

**Published:** 2025-05-02

**Authors:** T. Stead, A. P. Blaber, D. N. Divsalar, D. Xu, K. Tavakolian, J. Evans, R. Billette de Villemeur, M-P Bareille, A. Saloň, B. Steuber, N. Goswami

**Affiliations:** ^1^ Department of Biomedical Physiology and Kinesiology, Simon Fraser University, Burnaby, BC, Canada; ^2^ Biomedical Engineering Program, University of North Dakota, Grand Forks, ND, United States; ^3^ Biomedical Engineering, Retired, University of Kentucky, Lexington, KY, United States; ^4^ Institut de Médecine et de Physiologie Spatiales, Toulouse, France; ^5^ Gravitational Physiology and Aging Research Unit, Division of Physiology and Pathophysiology, Otto Löwi Research Center of Vascular Biology, Immunity and Inflammation, Medical University of Graz, Graz, Austria; ^6^ Center for Space and Aviation Health, Mohammed Bin Rashid University of Medicine and Health Sciences, Dubai, United Arab Emirates; ^7^ Health Sciences Division, Innlandet University College, Innlandet, Norway

**Keywords:** short-arm human centrifuge, presyncope, cardiovascular, heart rate, blood pressure

## Abstract

**Introduction:**

Exposure to microgravity results in physiological deconditioning, including orthostatic intolerance. Artificial gravity (AG) from short-arm centrifuges is being tested in ground-based studies to counter these effects. Orthostatic tolerance testing with centrifuges before and after these spaceflight analogs could determine the efficacy of an AG countermeasure to orthostatic tolerance. However, there has not been an investigation on how long before analog testing AG orthostatic tolerance data would remain valid for such a study.

**Methods:**

A secondary analysis of two experiments involving AG orthostatic tolerance testing (starting at 0.6 for females and 0.8 Gz for males and increased by 0.1 Gz every 3 minutes until presyncope) conducted 7 months apart at MEDES revealed 4 male and 3 female participants who had taken part in both.

**Results:**

Comparisons of participants’ time to presyncope between the two tests using Lin’s concordance correlation coefficient (LCCC) showed a significant relationship in time to presyncope between the two test dates (LCCC = 0.98) for males but not for females (LCCC = −0.64). While the cardiovascular data from one female was unusable, the mean heart rate responses to increasing artificial gravity during the orthostatic tolerance procedure showed a strong linear correlation between the two tests for all other participants (all p < 0.008). The LCCC heart rate changes with centrifuge level varied across male participants from 0.61 to 0.97, suggesting that the high LCCC for time to presyncope was achieved with varied HR baselines between the two test dates.

**Discussion:**

These findings indicate that time-to-presyncope tests may remain valid up to 7 months after the testing date for males. We highly recommend further study with larger numbers of male and female participants.

## Introduction

Long-term spaceflight, such as that seen with crewed missions on the International Space Station (ISS), introduces the challenge of physiological deconditioning within the human body which has been shaped by life on Earth. This deconditioning takes place across multiple body systems and includes the loss of muscle and bone mass, increased risk of thrombo-embolism, and the development of orthostatic intolerance (Blaber et al., 2011; Goswami et al., 2012; Goswami et al., 2013; Goswami, 2017; Goswami et al., 2019c; Harris K. et al., 2022; Harris K. M. et al., 2022; Rittweger et al., 2015). Orthostatic tolerance refers to the ability to maintain cerebral perfusion while standing, thereby maintaining oxygen delivery via blood flow to the brain. These adaptations that occur in the microgravity environment present as disadvantages and have disabling effects upon the subsequent return to Earth’s gravity. Since the establishment of a permanent presence in space with the ISS, scientists have spent much time in developing and perfecting countermeasures such as pharmaceuticals, nutrition, exercise prescription, lower body negative pressure (LBNP) (Goswami et al., 2019a; Goswami et al., 2019b; Goswami, 2023), and artificial gravity (AG) (Evans et al., 2018; Laing et al., 2020; Tanaka et al., 2017; Winter et al., 2018).

When conducting AG studies using a short-arm human centrifuge (SAHC), typically the research team conducts a time-to-presyncope test to determine the gravitational-load tolerance limit of the individual (Evans et al., 2015; Goswami et al., 2015). This test is primarily conducted to find the upper limit of gravitational forces the person will experience before losing consciousness. The prescription of AG used afterward is usually based on this time-to-presyncope test. As such, the presyncope test is conducted first, normally a few weeks before the targeted study intervention is to be carried out. For this reason, it is important to understand how long the time-to-presyncope test remains valid for screening participants. The repeatability of presyncope tests has been explored in other spaceflight countermeasures, such as LBNP (Goswami et al., 2009), which did not show a difference in heart rate, mean arterial pressure, total peripheral resistance, cerebral artery blood flow velocities, and cerebral oxygen saturation between exposures separated by 4 weeks (Kay and Rickards, 2015). Therefore, the purpose of this study was to investigate the repeatability of SAHC time-to-presyncope test protocols based on heart rate responses for healthy males and females spaced 7 months between exposure to the same presyncope protocol.

## Methods

### Participants

Secondary analysis of data from volunteers who took part in two independent centrifuge research projects with the same presyncope protocol were used. In both projects, the presyncope test was used to determine participants’ orthostatic tolerance before implementation of any test procedures. A review of participants from the two studies found four males and three females who took part in both AG studies. They ranged in age from 27 to 34 (31 ± 2.9, mean ± SD) years with an average height of 170 ± 7.1 cm and an average weight of 70.2 ± 5.0 kg.

Selection criteria for both projects were the same. The recruited participants were healthy, un-trained, non-obese, non-smoking individuals free of chronic illness or acute infectious disease, ENT, orthopedic, cardiovascular, and neurological disorders, particularly orthostatic hypotension, and vestibular disorders. They were also free of alcohol or substance dependence and did not require medical treatment. Participants also underwent medical examination, including screening by a physician, urinalysis, and an exercise stress test. If all medical criteria were met, the participants underwent an orientation session with the SAHC, where they were centrifuged at 1.0G at the foot for 10 minutes. Those participants without vestibular or cardiovascular disturbances were then enrolled in the study.

Presyncope data were collected from two separate sessions using the same protocol in July 2021 and February 2022, using the SAHC at the MEDES Space Clinic in Toulouse, France.

Ethical approval for all research was obtained from the *Comité de Protection des Personnes/CPP Sud-Ouest Outre-Mer I* and the *Agence Française de Sécurité Sanitaire des Produits de Santé* for each aspect of the study and scientific protocols. Research associated with our analysis was approved by the Office of Research Ethics at Simon Fraser University. A written informed consent was signed by each participant.

### Equipment for AG and data collection

The device used to collect heart rate (HR) during both protocols was a three-lead ECG (Philips Monitor IntelliVue) using the Lead II configuration of electrode placement. Blood pressure was recorded from a non-invasive photoplethysmograph (BMEYE, Amsterdam, Netherlands) placed on the index finger of the participant’s right hand. The SAHC device used in both the July and February protocols was located at the MEDES Space Clinic in Toulouse, France (European Space Agency).

### Protocol

**FIGURE 1:**
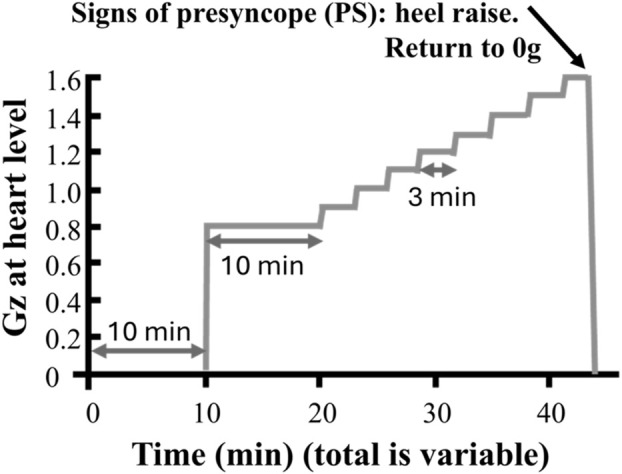
Example of a time to presyncope protocol. Participants were instrumented for heart rate and blood pressure monitoring while supine in the nacelle of the short-arm centrifuge. Following a 10-min rest period while systems were checked, centrifugation started with a 10-min baseline at 0.8 g (0.6 g for female participants) at the level of the heart after which a ramped of 3-min 0.1 g increments was applied until onset of presyncope (based on Goswami et al., 2015). At the onset of presyncope, participants were told to do a heel raise to activate venous return via the skeletal muscle pump, while the centrifuge was slowed to 0 g through a controlled stop.

The protocol for the time-to-presyncope test (Figure 1) in both studies had the participants lie supine in the centrifuge with baseline level 0 G for 10 minutes, followed by 10 minutes 0.6 G for the female participants and 0.8 G for the male participants at the level of the heart (detailed in Goswami et al., 2015; Evans et al., 2015). The acceleration of the SAHC was increased by 0.1 G every 3 minutes, continuing until the participants showed signs or symptoms of presyncope—defined using any of the following criteria: i) a drop in systolic blood pressure (SBP) below 80 mmHg or by a drop ≥20 mmHg/min, ii) a drop in diastolic blood pressure (DBP) ≥10 mmHg/min, iii) a drop in heart rate ≥15 bpm, iv) the occurrence of light-headedness, visual disturbances, nausea, clammy skin, or pallor skin, and/or v) at the request of the participant (Gao et al., 2008; Hinghofer-Szalkay et al., 2011)—or until the cardiac output dropped to half of the outset value. Real-time interpretation of the ECG for heart rate, and the blood pressure signal for systolic, diastolic, and mean arterial blood pressure along with Windkessel model (Wesseling et al., 1993) derived cardiac output were displayed solely for medical monitoring during the operation of the SAHC and not recorded. Participants did not change lifestyle habits between the two studies.

### Data analysis and interpretation

The ECG and blood pressure waveforms were exported from their respective devices and recorded simultaneously at 1,000 Hz using a National Instruments USB-6218 16-bit data capture equipment and LabVIEW 2013 software (National Instruments Inc, TX, United States). Beat-by-beat analysis of these data was performed off-line. Unusable data (raw data from beats in which the mean could not be reliably calculated, either due to noise or lack of signal) were removed and the time series adjusted by interpolating new values from the two valid points surrounding the excluded segment.

Statistical analysis of cardiovascular responses was performed using JMP® Version 16 (SAS Institute Inc., Cary, NC, United States). Baseline cardiovascular measurements were compared between the two test dates using paired Wilcoxon on ranks method.

The Lin’s concordance correlation coefficient (Lin et al., 2002) analysis was performed in Microsoft Excel to determine the repeatability of time to presyncope using the eight participants as well as for each sex. This procedure was also used to determine the repeatability of heart rate responses to changes in centrifuge g levels between the two dates. The mean HR at each g-level stage of the centrifuge protocol in both time-to-presyncope tests was determined from the ECG using MATLAB (MathWorks Inc., United States).

Secondary comparisons of time to presyncope and individual heart rate changes with g level were conducted using a linear regression function (JMP® Version 16).

## Results

The ECG and blood pressure signals from participant 110 were not usable. As a group, excluding participant 110, there were no significant and consistent differences in cardiovascular measurements between the two test dates (July 2021 vs. February 2022) in baseline HR (82 ± 4 vs. 86 ± 3 bpm, p = 0.38), SBP (143 ± 7 vs. 156 ± 9 mmHg, p = 0.58), or DBP (76 ± 3 vs. 90 ± 7 mmHg, p = 0.093), although there were varied individual responses with some showing large changes either increasing or decreasing from July 2021 (Figure 3).

**FIGURE 2:**
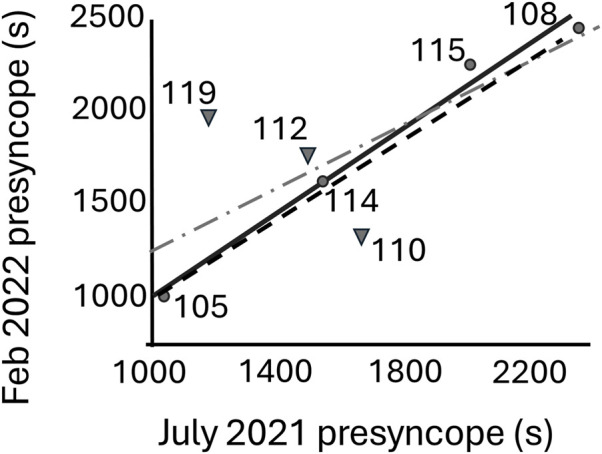
Linear regression fit line of time-to-presyncope test (s) for July 2021 and February 2022 for only the four male participants (circles, solid line) and with all the participants (addition of three females (triangles, dash-dot line). The line of concordance is shown as a dashed line.

A comparison of the time to presyncope from July 2021 and February 2022 gave an LCCC of 0.73 using the eight participants. This value increased when only male participants were included (LCCC = 0.98) and reduced with only female participants (LCCC = −0.64). The line of concordance is shown in Figure 2.

A comparison of the time to presyncope between the two study dates had a linear relationship for the group of men and women (n = 8) (Figure 2, dashed line) (Equation 1).

$${\text{Feb}}_{2022}=\left(394\pm 518\right)+\left(0.84\pm 0.31\right)\cdot {\text{July}}_{2021};\;\left(r^{2}=0.51,p=0.043\right)$$ (1)

However, the female participants (n = 3) did not show any significant relationship (r^2^ = 0.78, p = 0.22), whereas the male participants exhibited a strong linear relationship (Figure 2, solid line) between the two dates (Equation 2).

$${\text{Feb}}_{2022}=\left(-114\pm 179\right)+\left(1.11\pm 0.11\right)\cdot {\text{July}}_{2021};\;\left(r^{2}=0.98,p=0.008\right)$$ (2)

The concordance (LCCC) between the average HR at each level of centrifugation until presyncope was compared between the two test dates for each participant (Table 1) with individual lines of concordance lines provided in Figure 4. Three of the six participants had LCCC values greater than 0.95.

**TABLE 1:** Individual regression results (Figure 4) from the comparison of average HR of February 2022 with July 2021 and Lin’s concordance correlation coefficient (LCCC, Lin et al., 2002).

Sex	Participant	Intercept	HR slope	HR r^2^ adj	HR p-value	LCCC
Male	105	−6.0	1.09	0.99	0.003	0.97
	108	6.5	1.11	0.99	0.0001	0.61
	114	13.7	1.03	0.93	0.0003	0.77
	115	−10.9	1.13	0.95	0.0001	0.95
Female	112	−0.8	1.03	0.99	0.0001	0.99
	119	20.4	0.77	0.91	0.008	0.87

The linear relationship between the average HR at each level of centrifugation until presyncope was compared between the two test dates (Figure 4) using regression analysis. The HR over the two separate tests were found to be highly correlated with the minimum r^2^ of 0.91 (Table 1). All slopes were significantly greater than 0 (all p < 0.008). The average slope of the regressions was not significantly different from 1 and the average intercept not different from 0.

Five of the eight selected participants had their tests runs at the same time of day (morning or afternoon) on the two test dates. However, three participants (115, 112, and 119) did not. Initially, participants 115 and 119 underwent their tests in the afternoon, while participant 112 had their first test in the morning.

## Discussion

We examined the repeatability of the time-to-presyncope testing for SAHC which had not previously been studied. Our analysis revealed that the time-to-presyncope between the two test dates had a high degree of concordance (LCCC>0.95) and were highly linearly correlated in the males. Female participants’ times to presyncope, however, had a low LCCC (>0.7) and were not significantly linearly correlated between the two test dates. HR changes with Gz regressions were highly correlated in both males and females, with the slopes not different from one and intercepts not different from zero. However, the LCCC values ranged dramatically between male participants with two showing low concordance (<0.8), but not the females (LCCC>0.85). This may indicate that differences in cardiovascular baseline did not affect male presyncope time outcomes.

Overall, the SAHC presyncope test with males was repeatable after 7 months. While this observation may not be conclusive in the light of the limited number of male (n = 4) and female participants (n = 3), several factors could contribute, including sex and seasonal differences.

**FIGURE 3:**
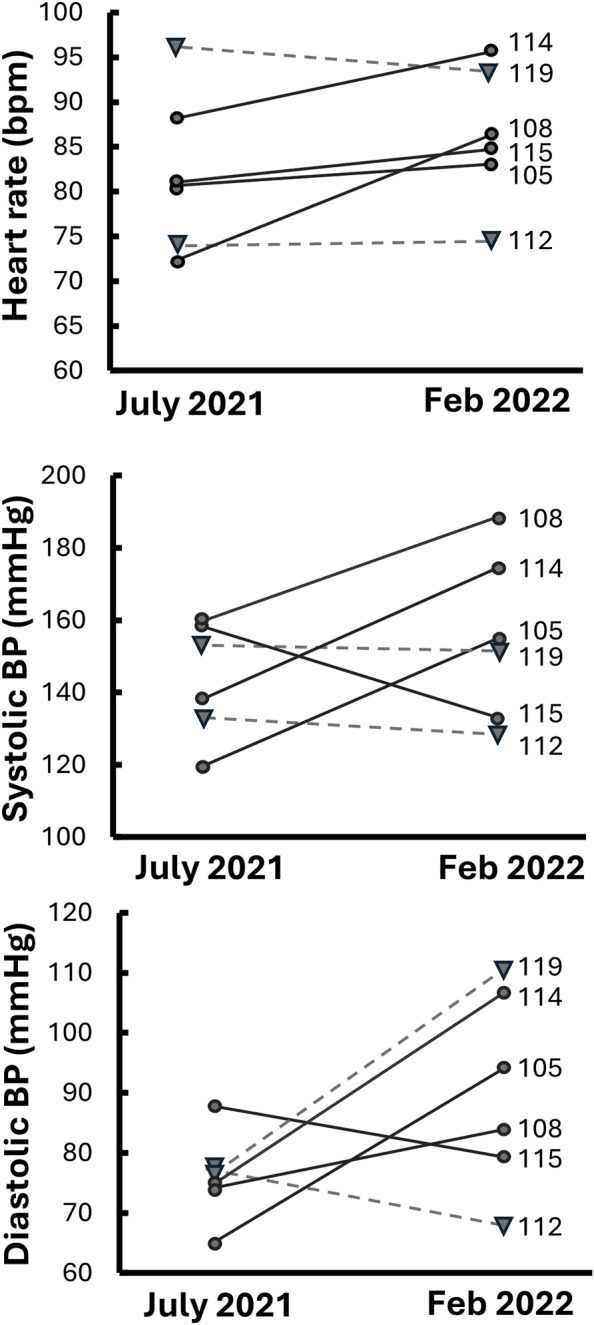
Individual cardiovascular changes for participants between July 2021 and February 2022. Solid lines and circles are male participants and dashed lines with triangles are female participants.

**FIGURE 4:**
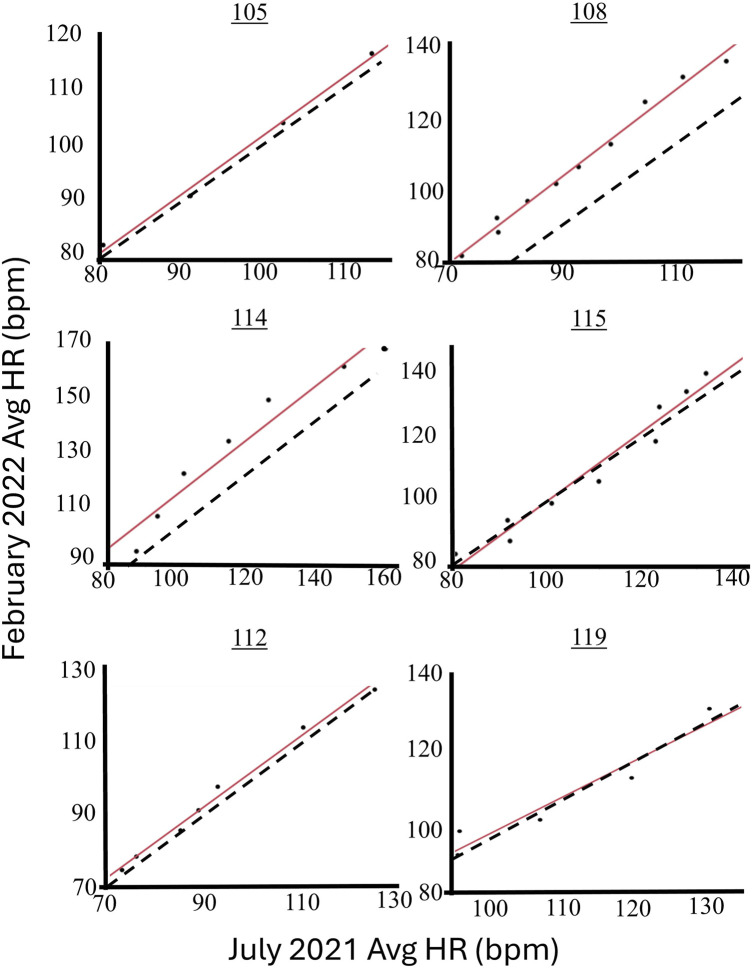
Linear regression fit lines (solid) for the HR responses of four male and two female participants where each data point represents the average HR for each increment of AG during the presyncope test for comparison of the July 2021 and February 2022 tests. The line of concordance is shown as a dashed line.

Previous literature has shown significant differences in the regulation of blood pressure and autonomic responses to orthostatic stress between the sexes, especially during hypovolemia, a major result of true space flight as well as models of spaceflight, like bedrest, immersion and hypovolemia (Evans et al., 2018; Goswami et al., 2019b; Patel et al., 2016; Shankhwar et al., 2023). In particular, participant 119 exhibited a much higher baseline DBP in February 2022 compared to July 2021 (110.57 vs. 74.73 mmHg)—and participant 105 displayed a similar pattern (Figure 3)—which suggests enhanced vasoconstriction in February 2022. This participant’s HR also increased slower in February 2022 than in July 2021 in response to increments of AG (Table 1; Figure 4), which could be attributed to greater vasoconstriction. As a result, participant 119 may have achieved longer presyncopal time in February 2022 compared to July 2021 (1927 vs. 1,201 s). While women typically respond to orthostatic stress with elevated HR (Arzeno et al., 2013; Mark et al., 2014), changes in the involvement of vasoconstriction of participant 119 across two test sessions could be a seasonal factor with reduced vascular function in the winter (Gordon et al., 2024). Menstrual phase has also been shown to affect hemodynamic responses (Shankhwar et al., 2024). Three of the participants were tested at a different time of the day, including 119, but the other two, one male and the other female, had no discernable difference in presyncope time. However, since the sample size is small, future studies on the reliability of presyncope testing should maintain consistent timing of tests and include factors like sex and environment.

Like LBNP and tilt testing (Ector et al., 1998; Güell et al., 1990; Benditt et al., 1996), human centrifugation can be used to determine and improve an individual’s orthostatic tolerance, (Goswami et al., 2015; Evans et al., 2015; Evans et al., 2004; Stenger et al., 2007). For comparison’s sake, LBNP tolerance has been shown to be repeatable up to 1 year for stepwise tests, and anywhere from one to 4 months for continuous ramp tests (Convertino, 2001; Kay and Rickards, 2015). Further, 90% reproducibility in tilt table testing has been shown for up to a week between tilt tests in a study using adult men and women (Grubb et al., 1992). Our results indicate that stepped centrifuge time-to-presyncope test can also be a valid screening tool for AG investigations that occur within 7 months from the test date.

Repeated human centrifugation has been shown to be an effective tool for increasing orthostatic tolerance in both men and women (Goswami et al., 2015; Evans et al., 2015; Evans et al., 2004; Stenger et al., 2007) but more effective in men than women (Stenger et al., 2007; Evans et al., 2015). This difference has only barely been studied and may provide information concerning basic sex differences in response to gravity. The repeatability of g tolerance in individuals implies a genetic influence on this variable, while the increase in g tolerance with repeated AG exposure indicates that the genetic influence can be modified. Establishing criteria to define a clear g tolerance limit will therefore be necessary to: 1) Distinguish individual tolerance levels to classify subjects’ performance, 2) determine effects of repeated AG exposures, 3) determine effects of other countermeasures (exercise, for example), 4) determine effects of deconditioning (space flight, bed rest, immersion, hypovolemia), and 5) determine gender, aging and inactivity effects.

### Limitations

Limitations of this study mainly lay in the small sample size of 4 male and 3 female participants that qualified for this investigation. However, we believe that this exploratory pilot study lays the foundation for carrying out bigger epidemiological studies in larger populations. These novel data also advance the literature related to AG-induced presyncopal times.

## Conclusions and future directions

As AG-induced presyncopal times were highly repeatable over 7 months in five of the seven participants tested, our results suggest that presyncope tests completed using SAHC-induced AG can be used to compare orthostatic tolerance pre- and post-exposure to interventions looking to improve orthostatic intolerance. However, with the small sample size and possible effects of sex and environmental factors, caution is required.

Tolerance for gravity diminishes during exposure to weightlessness, whether that be exposure to bedrest, water immersion or to spaceflight and a clear test for this tolerance needs to be developed. The criteria for this test will be how long its effects will last and how often it can be administered without significantly influencing the results. The present study indicates that AG tolerance time is a promising candidate for such a test in men that can be spaced as much as 7 months apart. Results from women, however, indicate that this test does not appear to be valid for women and that women’s response to AG exposure may be different from men’s. An effect that needs to be explored in future studies.

Finally, results of this study advance the literature related to orthostatic intolerance. Our results show the feasibility of stepped SAHC presyncope testing to determine an individual’s orthostatic tolerance limit. Upon further validation, this test will provide a gravity-based tool to assess orthostatic intolerance, itself a measure of a prospective astronaut’s fitness for spaceflight. Additionally, the presyncope test plays a crucial role in individualized centrifugation training which can be used as a countermeasure against orthostatic intolerance. In this training, the AG limit is determined from the maximum AG level at presyncope (Goswami et al., 2015). With orthostatic intolerance being a growing problem in geriatrics (Goswami, 2017; Goswami et al., 2017; Sachse et al., 2019; Trozic et al., 2019), an individualized training protocol could be an effective diagnostic tool considering the large, individual differences in the cardiovascular tolerance limits of seniors, but those studies are yet to be done.

## Data Availability

Data will be shared upon request if it conforms to the original conditions of ethical approval. Requests to access the datasets should be directed to Andrew Blaber, andrew_blaber@sfu.ca.
